# Deep learning empowered breast cancer diagnosis: Advancements in detection and classification

**DOI:** 10.1371/journal.pone.0304757

**Published:** 2024-07-11

**Authors:** Jawad Ahmad, Sheeraz Akram, Arfan Jaffar, Zulfiqar Ali, Sohail Masood Bhatti, Awais Ahmad, Shafiq Ur Rehman

**Affiliations:** 1 Faculty of Computer Science & Information Technology, The Superior University, Lahore, Pakistan; 2 Intelligent Data Visual Computing Research (IDVCR), Lahore, Pakistan; 3 Information Systems Department, College of Computer and Information Sciences, Imam Mohammad Ibn Saud Islamic University (IMSIU), Riyadh, Saudi Arabia; 4 School of Computer Science and Electronic Engineering (CSEE), University of Essex, Wivenhoe Park, Colchester, United Kingdom; Al-Nahrain University, IRAQ

## Abstract

Recent advancements in AI, driven by big data technologies, have reshaped various industries, with a strong focus on data-driven approaches. This has resulted in remarkable progress in fields like computer vision, e-commerce, cybersecurity, and healthcare, primarily fueled by the integration of machine learning and deep learning models. Notably, the intersection of oncology and computer science has given rise to Computer-Aided Diagnosis (CAD) systems, offering vital tools to aid medical professionals in tumor detection, classification, recurrence tracking, and prognosis prediction. Breast cancer, a significant global health concern, is particularly prevalent in Asia due to diverse factors like lifestyle, genetics, environmental exposures, and healthcare accessibility. Early detection through mammography screening is critical, but the accuracy of mammograms can vary due to factors like breast composition and tumor characteristics, leading to potential misdiagnoses. To address this, an innovative CAD system leveraging deep learning and computer vision techniques was introduced. This system enhances breast cancer diagnosis by independently identifying and categorizing breast lesions, segmenting mass lesions, and classifying them based on pathology. Thorough validation using the Curated Breast Imaging Subset of Digital Database for Screening Mammography (CBIS-DDSM) demonstrated the CAD system’s exceptional performance, with a 99% success rate in detecting and classifying breast masses. While the accuracy of detection is 98.5%, when segmenting breast masses into separate groups for examination, the method’s performance was approximately 95.39%. Upon completing all the analysis, the system’s classification phase yielded an overall accuracy of 99.16% for classification. The potential for this integrated framework to outperform current deep learning techniques is proposed, despite potential challenges related to the high number of trainable parameters. Ultimately, this recommended framework offers valuable support to researchers and physicians in breast cancer diagnosis by harnessing cutting-edge AI and image processing technologies, extending recent advances in deep learning to the medical domain.

## Introduction

Breast cancer is a significant health concern, especially in Asia, where diverse factors contribute to its prevalence. It imposes emotional, physical, and financial burdens on individuals and communities, necessitating global collaboration for early detection, improved healthcare, and tailored treatments. In 2023, an estimated 300,590 new cases and 43,170 deaths from breast cancer are projected in the United States [1]. Early detection and de-stigmatization are crucial for reducing mortality and promoting mental well-being [2]. The age and presentation differences between Asian and Western women with breast cancer raise questions about disease characteristics, emphasizing the need for research and awareness. The complex nature of breast cancer, with diverse subtypes and treatment responses, emphasizes the need for personalized treatment strategies. Public education on self-exams, mammograms, and a healthy lifestyle is crucial for prevention and early detection [3]. The evolution of medical imaging, from X-rays to modern modalities like mammography, ultrasonography, CT scans, MRI, and digital radiography, has significantly influenced cancer diagnosis and research [4]. These technologies have enabled the acquisition of crucial medical images, which are then analyzed by radiologists, playing a pivotal role in the diagnostic process. Mammography remains the preferred and reliable method for breast cancer screening, especially in the early stages. Modern mammography equipment utilizes digital technology, reducing radiation exposure and ensuring safety [5]. Its effectiveness in early detection emphasizes the importance of promoting breast cancer awareness and regular mammogram screenings for women, particularly those at higher risk. Mammography requires precise positioning of the nipple in alignment with the lower edge of the pectoralis major muscle. Two key views, MLO (mediolateral oblique) and CC (craniocaudal) are utilized to capture comprehensive breast tissue images. The CC view focuses on inner breast tissue without the axillary tail and centers on the pectoralis major muscle, ensuring accurate breast examination [6]. Radiologists are skilled in identifying potential cancer risks by analyzing mammograms for abnormal areas with increased brightness, location, breast size, and fatty tissue density [7]. They emphasize concern when dense, white tumor masses are evident, as malignant tumors can change in shape. Benign tumors pose minimal risk, but vigilance is necessary for anomalies like calcifications, asymmetries, and structural deformations, often caused by artifacts. Various techniques, including digital mammographic screening and full-field digital mammography (FFDM), are employed from the CBIS-DDSM dataset.

**Fig 1:**
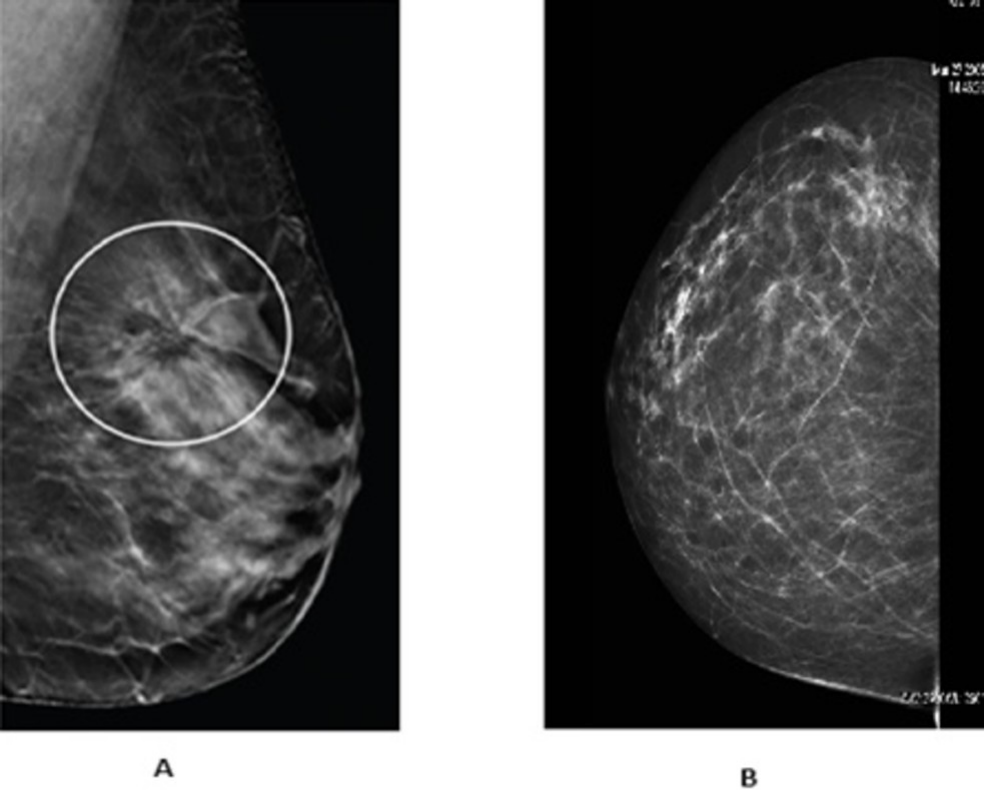
Breast mass (A) and Calcification (B) lesions.

**Fig 2:**
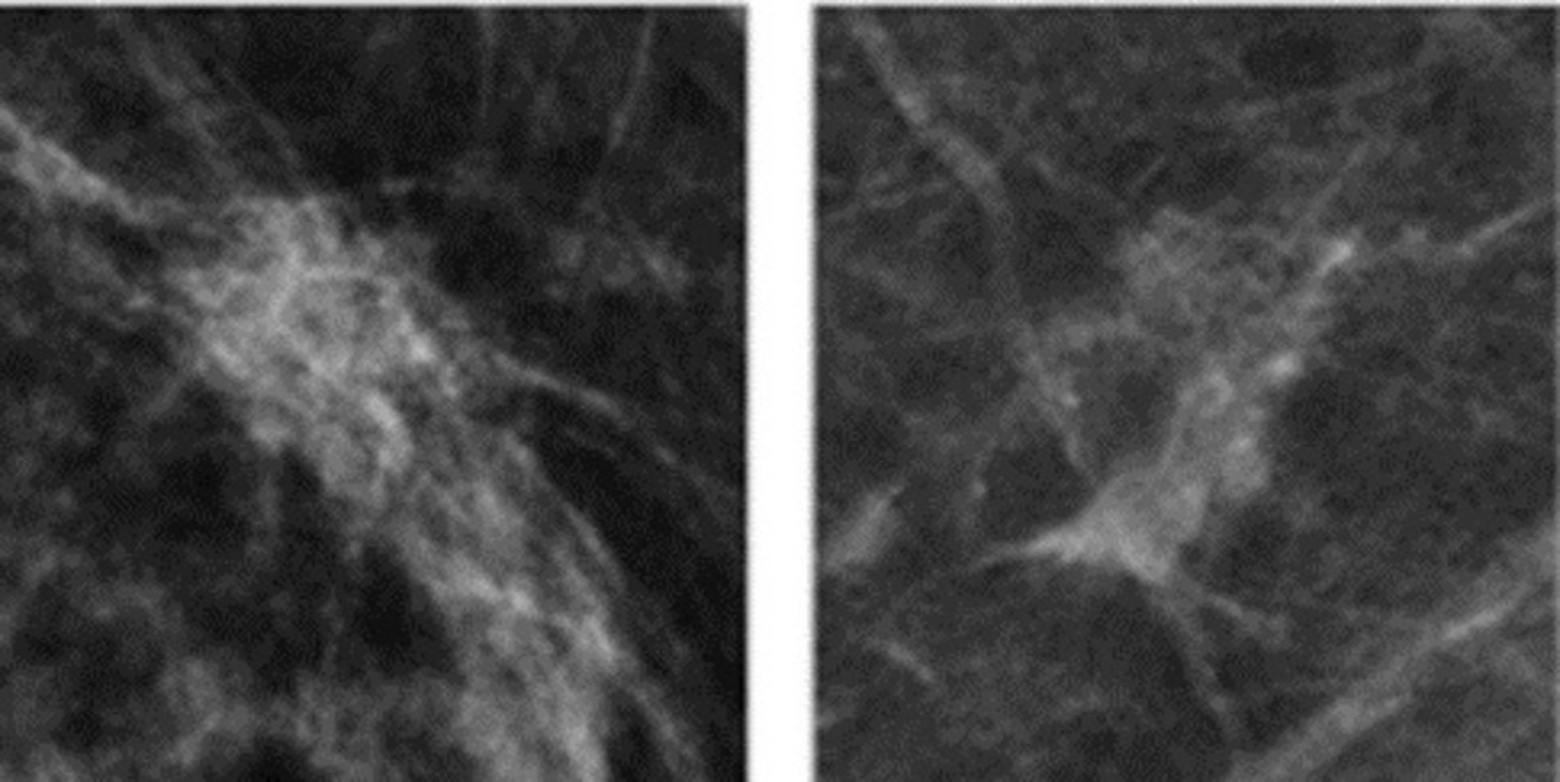
Breast architectural distortion lesions.

Mammography can identify one or more lesions inside the breast that vary in size and location. Radiologists routinely compare screening images over time to detect changes or confirm breast cancer-related symptoms. Breast mass lesions, which can be benign or malignant, are often identified through various methods, including mammography, biopsy, or MRI etc. Figs 1 and 2 showcase benign breast abnormalities, including calcifications and architectural distortion. Calcifications, visible as white patches and dots in mammograms, can sometimes be associated with ductal carcinoma in situ, though they are typically benign. Macro-calcifications appear as clear specks, while micro-calcifications, despite their small size, warrant closer attention due to their significance.

Architectural distortion in the breast, characterized by deformations without a visible tumor, is a common benign condition but can be a precursor to breast cancer. Detecting architectural distortion can be challenging, especially in 2D mammography, due to its shifting appearance, size, and position. Mammograms are valuable for categorizing breast abnormalities by isolating tumors from the background, offering cost-effective diagnoses. While these procedures often rely on manual interpretation by radiologists, computerized mammography analysis has the potential to enhance accuracy and effectiveness, aiding in the distinction between benign and malignant tumors, and ultimately improving diagnosis and treatment decisions. The study highlights the value of routine mammography exams in lowering mortality rates by identifying breast cancers early on before they have a chance to spread to other body parts or healthy tissues. As a result, radiology specialists review mammography every day to spot problematic lesions and evaluate any questionable breast tissue based on its location, traits, and shape [8]. This process continues to be costly and error-prone despite its importance and the increasing number of mammograms checked daily, underscoring the need for increased accuracy and dependability [9]. It is the job of radiologists to recognize worrisome lesions on breast mammograms during screening and to differentiate between other types of lesions, such as masses, calcifications, and other typical abnormalities. Physicians must then decide how to treat the tumor and determine its pathology diagnosis to determine if it is benign or malignant. As a result, computer-aided diagnostic (CAD) systems can offer a second opinion, assisting professionals in determining the possibility of breast cancer in general [10].

Recent developments in AI for computer vision have produced algorithms that have shown to be incredibly helpful to medical professionals. Particularly, these systems have proven their capacity to precisely detect, outline, and classify malignant lesions in a variety of medical imaging tasks, including mammography [11]. Conventional methods relied on straightforward image processing and machine learning techniques to extract hand-crafted and fundamental attributes with the aim of locating and identifying probable locations [12–14]. Cutting-edge deep-learning algorithms are emerging as substitutes for traditional tumor segmentation methods due to deteriorating accuracy and a high false positive rate. These new algorithms offer more sophisticated capabilities and address the limitations of conventional approaches by incorporating background tissue information and automating feature extraction for tumor delineation and classification in computer-aided diagnosis systems. [15]. Advanced machine learning methods, notably Convolutional Neural Networks (CNNs), are garnering attention in automated CAD systems and medical imaging for their proficiency in feature extraction and recognition, particularly in detecting subtle patterns associated with conditions like breast cancer. As computer processing power has grown, deep learning models have gained prominence for their ability to automatically extract comprehensive features from medical images, eliminating the reliance on prior knowledge or human feature engineering. [16]. This development has helped to improve automated system results while finding a crucial balance between their ability to recognize many lesions in a single mammogram and the accuracy of detecting these lesions [17,18].

Cutting-edge CAD systems leverage deep learning algorithms to provide real-time assistance to radiologists, enhancing early diagnosis and personalized treatment planning for breast cancer. While automated techniques improve detection accuracy, radiologists’ clinical expertise remains essential for prognosis and therapy decisions, underscoring the collaborative role of technology and human judgment in patient care. [19,20]. To reduce false positive and negative instances since breast cancers, CAD system performance must be generally improved. These features have led to widespread support for deep learning’s use in biomedical settings, particularly in CAD systems created for mammography [21,22].

Finding tiny breast cancers is fundamentally more difficult than finding larger, more advanced tumors. The use of sophisticated algorithms may aid in early detection, improve the chances of effective therapy, and ultimately result in better patient outcomes. During the past 20 years, deep learning has demonstrated its ability to address complicated challenges in the field of medical imaging by excelling in a range of computer vision tasks. As a result, we focus on mammography in particular tasks such tumor identification, breast lesion segmentation, and classification.

Mammography, introduced in 1913, has proven invaluable in early breast lesion detection, significantly reducing mortality rates through screening. Research emphasizes the role of Computer-Aided Diagnosis (CAD) systems, leveraging computer vision and AI, in automatically detecting anomalies in mammograms, aiding healthcare practitioners in medical imaging analysis. [23]. A technique for identifying cellular alterations in breast tissues that may differentiate between diseased and healthy situations was developed by Tavakoli et al. [24], Preprocessing procedures, a special block-based convolutional neural network (CNN) architecture, and the inclusion of a decision-making mechanism are all part of this method. This process creates a binary map that classifies pixels inside the defined region as either having anomalies or being within the normal range after the CNN has been trained. It is noteworthy that this method, when used on the MIAS database, has an amazing accuracy rate of 95%. Moon et al. [25] developed a computer-aided detection (CAD) system specifically for the purpose of identifying malignancies in their work. This system uses CNN architectures, multiple representations of the image content, and an image fusion technique. Combining these methods led to diagnostic performance metrics for the ensemble method of 91.10%, 85.14%, 95.77%, and 0.9697, respectively. In order to improve information propagation, the study used skip connections, ResNet, and DenseNet connections to solve issues such gradient vanishing and inter-layer transmission loss. It’s crucial to remember that in this investigation, B-mode ultrasonography (US) images were used to manually delineate tumors and surrounding tissue. It’s important to note, too, that depending on the operator, tumor shapes and regions of interest (ROIs) can vary. In order to identify and detect breast cancer, Khan et al. [26] created a ground-breaking method that makes use of deep learning. To improve classification accuracy, they used three different CNN architectures: ResNet, VGGNet, and GoogleNet. This solution, which also made use of data augmentation techniques, outperformed rival approaches by an astounding 97.525%. The study investigated a combination of human created features and features retrieved by CNNs in order to further hone the categorization process. The difficulties in image categorization, particularly when dealing with complicated histological images of breast cancer, have been efficiently handled by contemporary breakthroughs in artificial intelligence and image processing techniques. The classification of histological breast cancer images has benefited significantly from the shift from traditional hand-crafted features to features obtained from Convolutional Neural Networks (CNNs) trained on patch images. Importantly, CNNs reliably produce objective findings across different datasets by classifying data without largely relying on domain-specific expertise. Similar networks may likewise produce positive results in this area. Peng et al. published a novel approach for automated mass detection [27]. They combined the multiscale-feature pyramid network and the Faster R-CNN model. Using the CBIS-DDSM and INbreast datasets, our technique showed impressive true positive rates of 0.94 and 0.96, respectively. In their work Masni et al. [28] developed a computer-aided diagnosis system based on YOLO. When used with the DDSM dataset, this system has an accuracy rate of 85.52%. For deep learning applications in medical image processing, Haq et al. [29] proposed using a Convolutional Neural Network (CNN) model, specifically the DnCNN model, with an emphasis on breast imaging data for breast cancer diagnosis. Within a 30-minute processing window, their proposed DnCNN model performed superbly, obtaining an amazing accuracy rate of 79%. Vedalankar et al. [30] used three easily accessible databases CBIS-DDSM, DDSM, and mini-MIAS to solve the issue of class imbalance in mammography datasets. Their suggested strategy called for the classification of architectural distortion in mammograms using support vector machines and AlexNet. The results demonstrated the method’s superiority over conventional methods with a peak accuracy of 92%, a sensitivity of 81.5%, and a specificity of 90.83%. It’s crucial to remember that this study is constrained by its reliance on a rather small group of three databases. This demonstrates the necessity for additional validation using larger and more varied datasets to guarantee the robustness and generalizability of the strategy.

Alruwailia and Gouda, et al. [31] concentrated on utilizing deep learning models to improve diagnostic mammography procedures for finding breast cancer in their study. To discriminate between benign and malignant breast cancer cases, they used transfer learning with pre-trained models, notably ResNet50 and Nasnet-Mobile. They also used augmentation techniques to increase the amount of mammographic images in order to improve the system’s stability, avoid overfitting, and broaden the dataset. The study’s findings showed that their deep learning system on the MIAS dataset had an accuracy of 89.5% when using ResNet50 and 70% when using Nasnet-Mobile. Importantly, when utilizing MOD-RES + oversampling (for ResNet50) and Nasnet-Mobile, their deep learning-based strategy surpassed professional radiologists across a range of parameters, including overall accuracy, precision, recall, and F1-score. Comparative studies showed that the suggested strategy outperformed the existing models in the field of medical imaging, especially when working with small training datasets. This highlights the potential for deep learning approaches to improve the precision and effectiveness of diagnosing breast cancer early using mammography. According to Das et al. [32], early breast cancer identification is crucial for increasing women’s survival rates. In order to help radiologists correctly diagnose breast cancer, their research relies on computer-aided diagnostic (CAD) systems. They carry out a comparison analysis utilizing several criteria to compare deep CNN architectures that have been trained on various datasets with a newly proposed shallow CNN architecture. The work makes use of shallow CNNs that take advantage of distinguishable features and preprocessed mammography pictures. By enhancing well-known CNN models including VGG19, ResNet50, MobileNet-v2, Inception-v3, Xception, and Inception-ResNet-v2, they also investigate transfer learning. Notably, the accuracy rates for the DDSM and INbreast datasets for the shallow CNNs are 80.4% and 89.2%, respectively. Pre-trained CNNs, on the other hand, are more accurate, with rates of 87.8% and 95.1% for the same datasets. These findings demonstrate the potential of shallow CNN architecture and pre-trained CNN models for efficient breast anomaly detection and precise cancer diagnosis. Different image dimensions and quality may be to blame for the observed performance discrepancies between the CBIS-DDSM and INbreast datasets. While deep learning-based features can result in overfitting, the INbreast dataset has improved mammography quality. On the smaller INbreast dataset, however, fine-tuning parameters enhance model performance. Although cross-dataset evaluations are part of the research, they produce fewer promising outcomes than within-dataset testing. These results offer insightful information for upcoming study topics. It’s important to note that other clinical characteristics, such as medical history or regional variations, which could improve computer-aided approaches for early cancer diagnosis and individualized care, are not taken into account in this study. The study acknowledges transfer learning’s limits, particularly when natural image features fall short of accurately capturing the subtleties of medical imaging. In order to overcome this, the authors suggest that transfer learning from datasets in the specific medical domain may result in algorithms for breast cancer diagnosis that are more accurate. The suggested CNN models show superior information extraction from individual images as compared to training each CNN from scratch after being thoroughly examined using contemporary methods. Notably, the maximum level of feature extraction efficiency is attained by the Xception model, which incorporates depth-wise separable convolution for recovering obscured objects and optimizes ResNet principles. The comparative analysis demonstrates the updated Xception classifier’s superior performance in comparison to other models. With performance scores ranging from 0.87 to 0.91 for the CBIS-DDSM dataset and 0.91 to 1.00 for the INbreast dataset, the upgraded Xception classifier consistently outperforms previous techniques, exhibiting excellent efficacy in the identification of breast cancer. An advanced framework based on deep learning and machine learning techniques was developed by Trang et al. [33] in their study with the main goal of detecting breast cancer by merging clinical data and mammography images. 731 pictures from 357 women made up the dataset used in this study, which was used to train a model that could distinguish between benign and malignant tumors. To do this, the researchers developed models for support vector machines, random forests, gradient boosting machines, and artificial neural networks (ANN) using clinical data. In order to assess mammograms, they also used deep convolutional neural networks (CNN), such as X-ception, VGG16, ResNet-v2, ResNet50, and CNN3. The combined model has an area under the curve (AUC) of 0.88, a sensitivity of 89.7%, a specificity of 78.1%, and an overall accuracy of 84.5%, according to the study’s findings. Surprisingly, the combined model performed better than utilizing just mammography pictures, increasing accuracy from 72.5% to 84.5%. This study brought to light the benefits of combining clinical information with mammography pictures to improve the precision of breast cancer detection. The study concluded that the combination of clinical and imaging data could improve the capacity of machine learning and deep learning models in the detection of breast cancer, thereby opening up new paths for therapeutic applications in the future. In order to address women’s health issues related to breast cancer, The paper emphasizes the limitations associated with using mammograms for breast cancer diagnosis and admits the frequent lack of explainability and interpretability in these systems, despite the outstanding segmentation and classification abilities of deep neural network-based CAD systems. Both individuals and medical professionals may become less trusting as a result of this restriction. To close this gap, the suggested methodology blends CBR and deep learning to produce precise and understandable classifications, improving the accuracy and comprehension of breast cancer detection. In order to automate the segmentation of breast cancers, Hai et al. [34] were pioneers in the development of a network that includes multiscale picture features. This network received scores of 60.41% for Intersection over Union (IoU) and 76.97% for Dice on an independent dataset. In an experiment, Soulami et al. [35] used a thorough UNet model to concurrently recognize, segment, and categorize breast masses. Using the INbreast and DDSM datasets to evaluate segmentation ability, a stellar Dice score of 90.50% was attained. Shams et al. [36] created an end-to-end model, for instance, that smoothly incorporated Convolutional Neural Networks (CNN) with Generative Adversarial Networks (GAN). They also provided a graphic of this integrated strategy. Their main objective was to categorize mammograms as benign or malignant. In studies utilizing the DDSM dataset, they were able to get an accuracy rate of 89%, while using the INbreast dataset, they were able to acquire an astounding accuracy rate of 93.5%.

A deep learning-based computer-aided diagnostic (CAD) method for early breast cancer diagnosis was created by Hekal et al. [37]. Using CNN models with adjustable Otsu thresholding, they improved the extraction of TLR (Texture and Location Relationship) characteristics and increased the training process’ effectiveness. The mammography nodule images were divided into four groups by the CAD system using a support vector machine (SVM)-based classifier: Benign Calcification, Malignant Calcification, Benign Mass, and Malignant Mass. Utilizing the ROI CBIS-DDSM dataset, the study presented its findings. The CAD system successfully classified ROIs into these four classes with noteworthy accuracy, achieving an accuracy of 0.91 using the AlexNet model and 0.84 using the ResNet-50 model.

In order to enhance the classification outcomes of the MIAS dataset, Saber et al. [38] proposed a deep learning architecture with a primary focus on identifying and diagnosing breast cancer. The dataset underwent a number of preprocessing procedures, including the detection of cancerous areas, noise reduction, and contrast enhancement. They used approaches for data augmentation to improve the dataset. Notably, they improved mass-lesion classification by utilizing freezing and fine-tuning techniques. When compared directly to alternative models, the VGG16 model showed remarkably high diagnostic accuracy for breast cancer. With values of 98.96%, 97.83%, 99.13%, 97.35%, 97.66%, and 0.995, respectively, it earned remarkable metrics for overall accuracy, sensitivity, specificity, precision, F-score, and AUC when utilizing the 80–20 technique. With performance scores of 98.87%, 97.27%, 98.2%, 98.84%, 98.04%, and 0.993, the VGG16 model performed admirably. The above literature review suggests that feature extraction, detection, and classification tasks may not be sufficiently accurate or efficient for the present CNN-based approaches for breast cancer detection. To obtain the appropriate degree of precision, these procedures also appear to need more time and resources. This research endeavor has a strong emphasis on raising detection accuracy. Despite the use of complex models in prior studies, it is important to note that the data used in this study showed an unequal distribution. Our goal in this research is to provide a quick and effective breast cancer diagnosis tool.

## Proposed methods

An overview of the extensive architectural models and techniques utilized in a CAD system for the early detection of breast cancer is given in this part. To extract features, find anomalies, segment tumors, and classify them, it makes use of cutting-edge deep learning and computer vision methods. For thorough diagnostic support, the system fused YOLO detection, segmentation using Associated-ResUNets, and classification through AlexNet (BreastNet-SVM).

A. **Detection and identification**

The YOLO network was developed as a departure from the conventional sliding window approach, aiming to predict both bounding box locations and class probabilities for the entire image using a single CNN. This innovative design significantly reduces computational overhead. At the heart of YOLO’s architecture lies a fully convolutional neural network (FCNN), illustrated in Fig 4, which divides the image into grids and generates bounding boxes, class probabilities, and confidence ratings for each grid cell.

**Fig 3:**
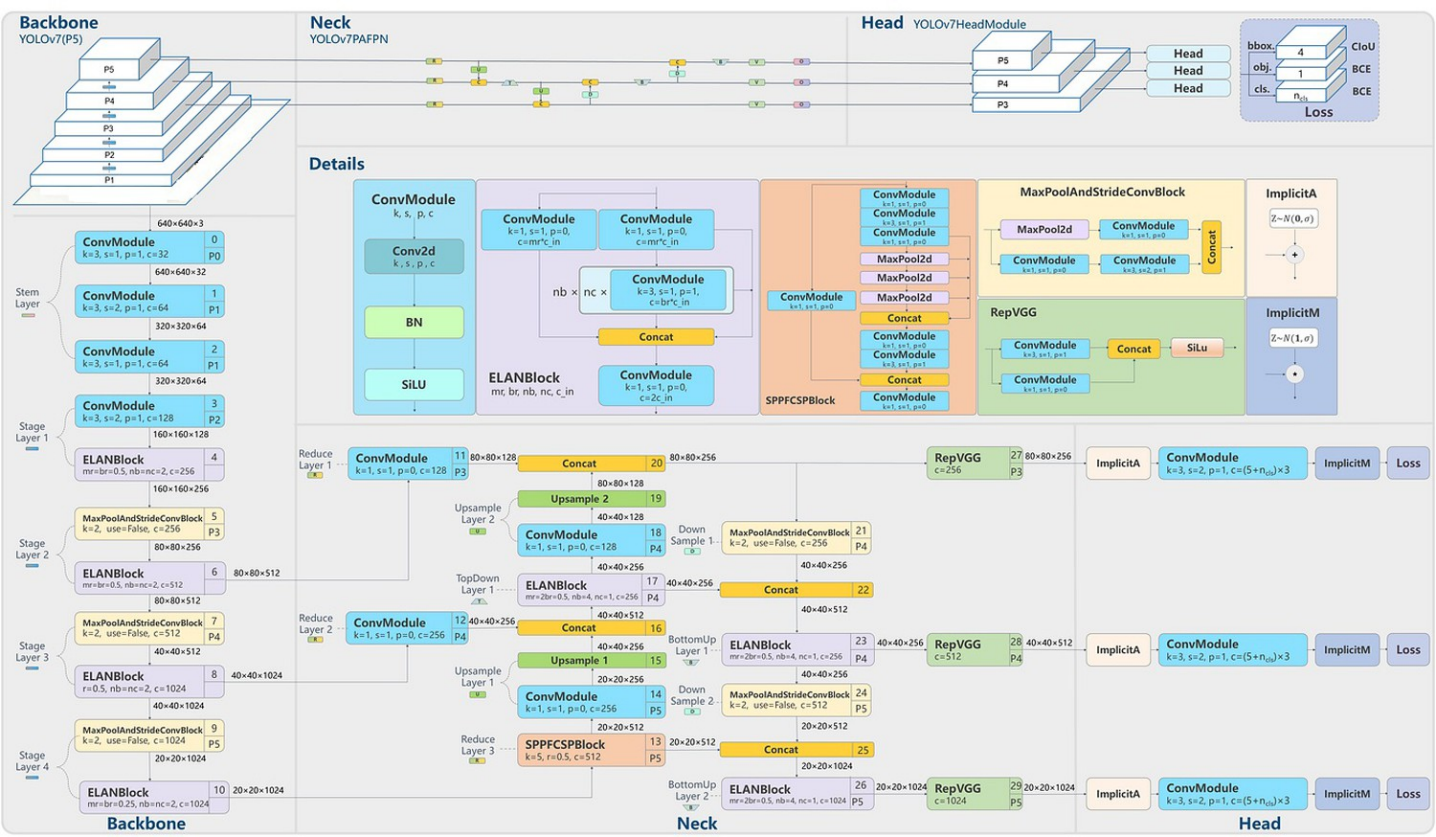
YOLO-V7 architecture [39].

We used YOLO-V7, the improved YOLO network’s seventh iteration, which was especially designed to improve object detection at various scales. The multi-scale feature extraction and detection method is used by YOLO-V7. As shown in Fig 3 [39], it first uses skip connections to address gradient vanishing problems in deeper network layers. Three fully connected layers that handle features extracted at various scales make up the detecting segment. The system uses anchor box theory to establish anchor boxes and fine-tunes them using a K-means method with whole images, both of which are inspired by the Faster-RCNNs model. The output matrices of multi-scale features are then arranged into grid cells and used along with these anchor boxes. The selection of boxes with scores over a predetermined threshold is made easier by this design, which also makes it simpler to calculate the Intersection over Union (IoU) percentages between ground-truth and anchor boxes. In order to ensure precise identification when both scores exceed a predetermined threshold, the model predicts confidence levels, probability distributions, and four offset values for each anchor box.

**Fig 4:**
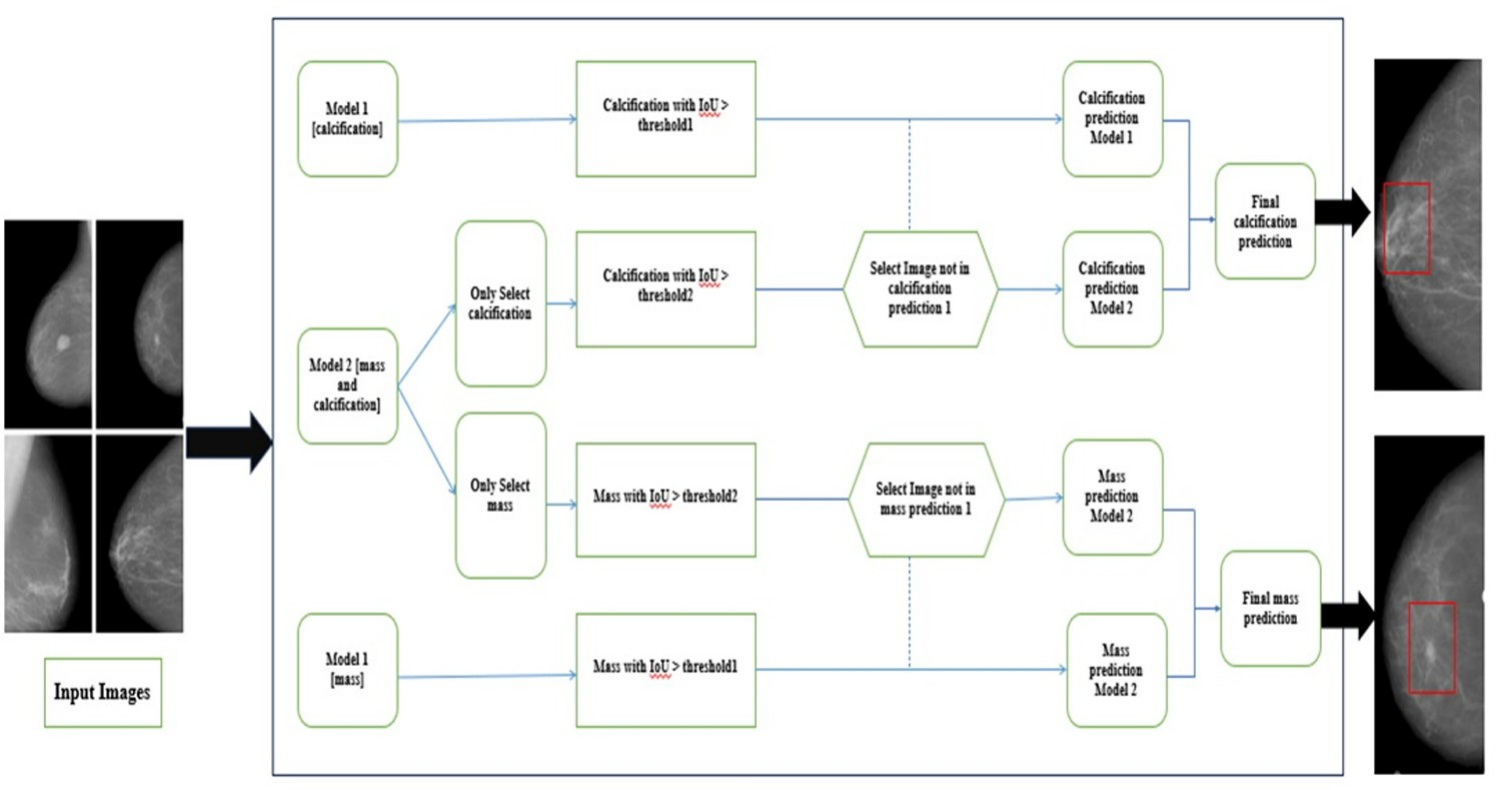
Flowchart for the fused models.

Our algorithm detects probable breast lesions within bounding boxes and assigns confidence scores, as discussed in the preceding section. This is consistent with the YOLO-based architecture shown in Fig 4. The model settings, input data, and YOLO’s classification method for identifying the lesion type (mass or calcification) all affect the confidence score. This lays the groundwork for enhancing prediction results. In this paper, we propose ranking the Intersection over Union (IoU) scores of various augmented images, including rotations and morphs, in order to prioritize the selection of precise predicted bounding boxes. This method aids in the selection of sample mammograms for accurate localization and classification of lesions. Additionally, to cut down on errors and improve overall performance, we advise merging predictions from various model implementations. These models undergo unique setup and training, such as Model-1 for Mass and Calcification independently and Model-2 for numerous classes. Following extensive testing, we develop customized fused models for calcification using Model-1(calcification) and for mass using Model-1(mass). The mass and calcification aspects of Model-2 considerably improve the general usability of the Model-1 models. Beginning with initial Mass predictions from Model1(Mass), our fusion technique concentrates on predictions with an IoU score over threshold1. After separating images with mass lesions using threshold2, we apply Model-2 (Calcification & Mass) to produce predictions. Mass Predictions 2 are defined as images that Mass Prediction 1 does not cover. Combining these two prediction groups yields the final Mass predictions shown in Fig 4. Calcification forecasts are made using a similar process. We employ threshold1 (0.44) and threshold2 (0.38) consistently throughout this fusion procedure since they have a history of producing promising outcomes.

To implement our strategy, we used a YOLO-based architecture. The core model was initially trained using a variety of configurations, each of which focused on a different class label, such as mass, calcification, or architectural deformation. To determine the projected bounding boxes with the best confidence scores for each iteration, we gathered a variety of augmented images, including the originals and rotational versions. This technique was developed to accurately identify the best images for the classification of certain mammograms and the precise diagnosis of breast abnormalities. we used a fused YOLO-model strategy to improve the results of our final forecast. By merging several forecasts, we hoped to lower overall error rates and increase the adaptability of models with various configurations. Model-2, built on YOLO, was set up for multi-class training including all three classes whereas Model-1 represented the YOLO base model created for a particular class. By analyzing both M-1 and M-2, the Fused Model was created to enhance overall detection performance. A new class label named "Normal" was also included to account for mammograms that came back normal during follow-up screening. Assuring the lack of anticipated bounding boxes, we employed the YOLO-based architecture trained on abnormal mammograms to forecast normal ones, permitting reliable categorization as "Normal." The most recent screening mammograms were used for the models’ creation and testing, which included examples of lesions with architectural deformation, calcification, or both. This all-encompassing strategy displays our dedication to improving and expanding the applicability of the YOLO-based paradigm.

B. **Segmentation**

**Fig 5:**
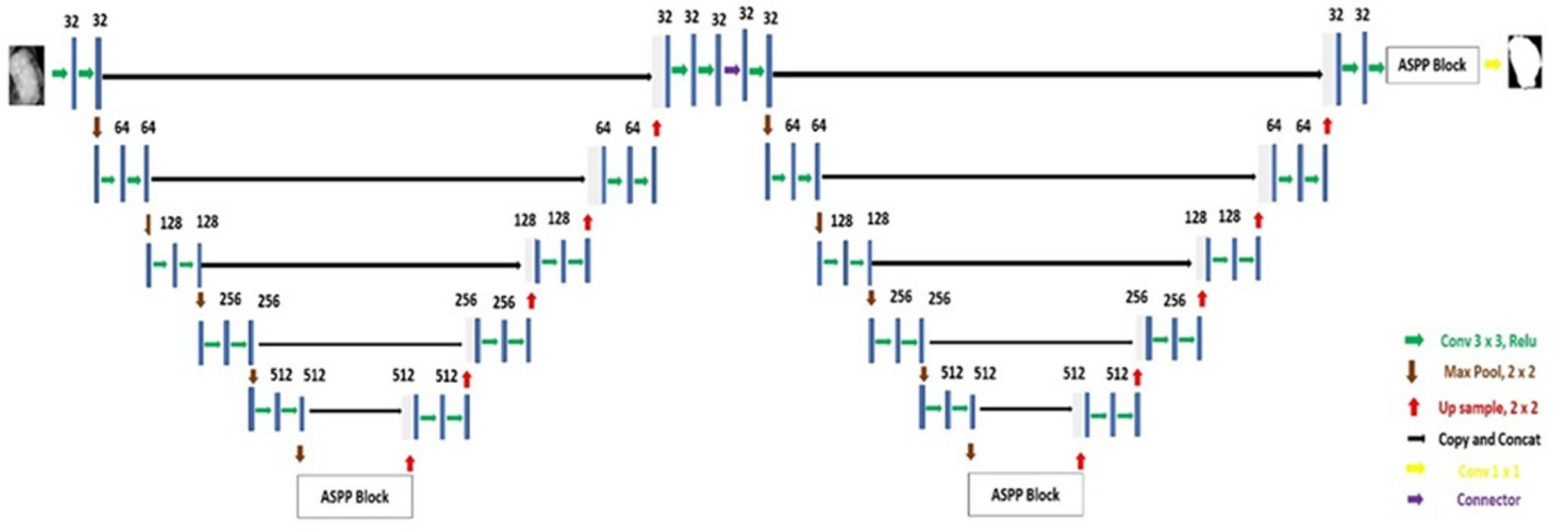
Architecture of Associated-ResUNets.

UNet, a prominent model in medical image segmentation, adopts an encoder-decoder structure inspired by FCN, omitting fully connected layers. Its symmetrical architecture comprises down-sampling and up-sampling paths, forming a "U" shape. UNet’s key innovation lies in integrating skip connections, vital for preserving spatial information lost during down-sampling. Inspired by this, the "Associated-ResUNets" architecture joins two UNets with additional skip connections to enhance information flow as shown in Fig 5. Each encoder block includes two convolution units followed by BN and ReLU layers, with the output undergoing max pooling before passing to the next encoder block. Customized skip connections between the first decoder and second encoder blocks recover decoded information, improving overall segmentation performance.

To facilitate smooth transitions between down-sampling and up-sampling pathways, the model employs an Atrous Spatial Pyramid Pooling (ASPP) block. This technique, utilizing "Atrous" convolution, widens the receptive field while maintaining resolution. The ASPP block integrates batch normalization layers and four 3 * 3 convolution layers with varying dilation rates, combined to generate multi-scaled features fed into a 1 * 1 convolutional layer. Following the initial UNet design, a second UNet with increased skip connections and insights from initial up-sampling is utilized. After activation with ReLU and normalization with a BN layer, the output of the preceding decoding block is merged with itself and used as input for the second UNet’s initial encoder block. Subsequently, the outputs of three encoder blocks’ maximum pooling methods are merged with the outcomes of preceding decoding blocks before down-sampling. The terminal encoding block of the second UNet is directed to the ASPP block, followed by a 1 x 1 convolutional layer and sigmoid activation layer to produce the final output mask. Additionally, the A-ResUNet model incorporates an attention block to fuse attention mechanisms with skip connections in encoder and decoder blocks. This attention block, accepting low-level data input, involves a transposed convolutional layer followed by ReLU activation, sigmoid activation, and transposed convolutional layers to produce an attention map. This map is multiplied with skip connection information to enhance segmentation accuracy. Finally, the decoder block receives input from this output to improve UNet’s segmentation capability across varied medical image sizes, with one typical convolution block replaced for optimization.

C. **Classification using a BreastNet-SVM**

**Fig 6:**
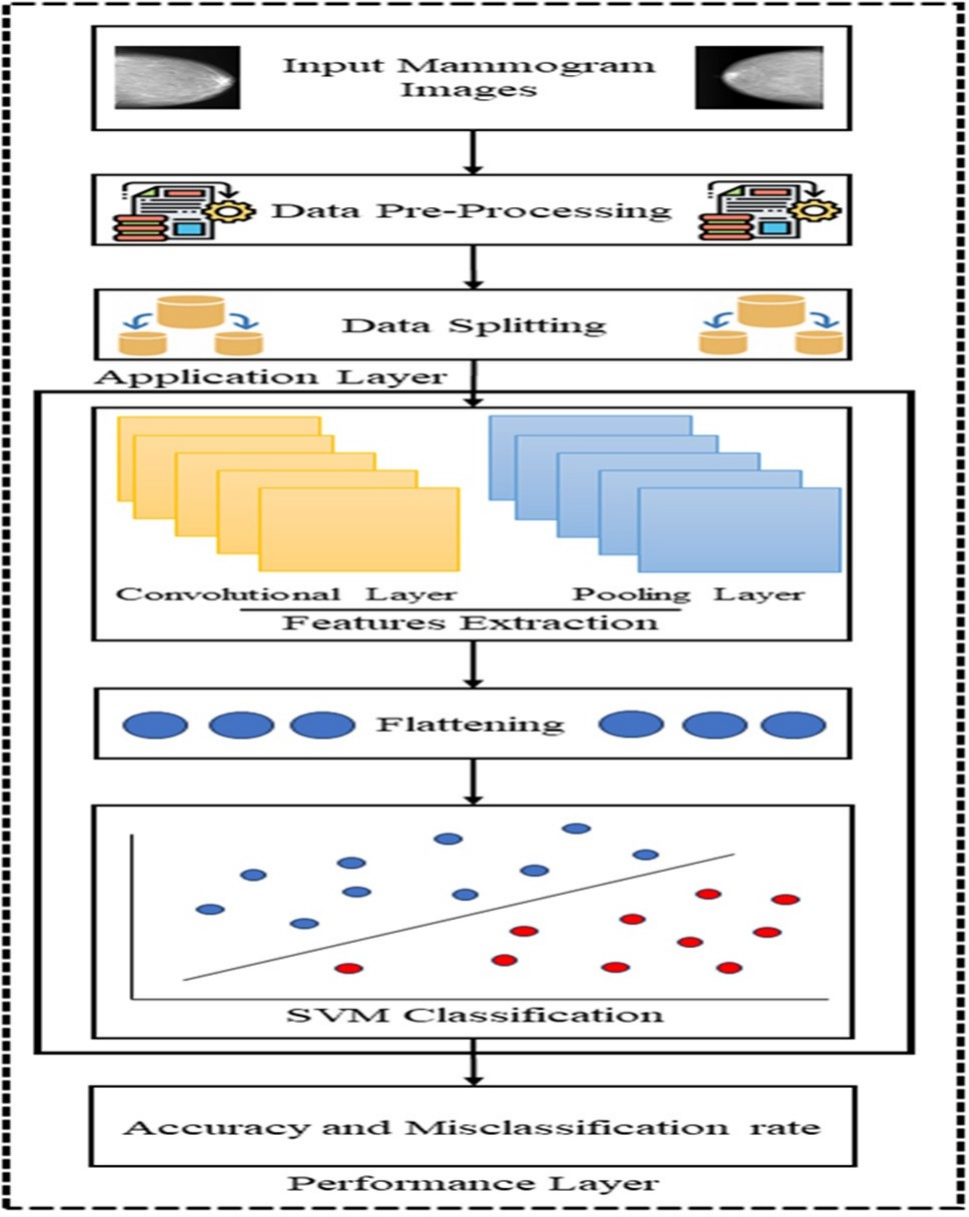
The Proposed BreastNet-SVM framework.

In this phase, we introduce a customized technique inspired by the architecture of AlexNet and its modified variants, forming the fundamental model termed BreastNet-SVM. Illustrated in Fig 6, this model encompasses training, validation, and testing phases, using the CBIS-DDSM dataset as the initial data source, comprising mammograms from individuals diagnosed with breast cancer. The data preparation stage involves enhancing data quality through preprocessing, including image transformations, noise removal, and outlier filtering. Subsequently, the meticulously processed data is divided into training, validation, and testing sets, with approximately 70% allocated for training and the remaining 30% for validation and testing. Notably, input patches can vary in size: 16 x 16, 32 x 32, or 48 x 48. The training dataset is composed of two main layers: the application layer and the performance layer. In the application layer, features are extracted using the modified convolutional neural network BreastNet-SVM, capturing significant information from input images for further processing. To optimize the model, three different optimization algorithms—Stochastic Gradient Descent (SGD), Adaptive Moment Estimation (Adam), and Root Mean Square Propagation (RMSprop)—are employed with specific hyperparameters, including a learning rate of 0.0001, 70 batches, and 150 epochs. The performance layer evaluates the precision and misclassification rate of the proposed BreastNet-SVM model.

In Convolutional Neural Networks (CNNs), the convolutional layer is an essential component that is in charge of identifying significant features in the input data. These layers carry out convolutional operations, represented by the symbol. They first apply a filter on the incoming image, though. It is common to refer to the result of this convolutional procedure as either an activation map or a feature map. Eq (1) depicts this convolutional operation visually.


$$A(i,j)=(X*Y)(i,j)=\sum_{\text{a}}\sum_{\text{b}}X(a,b)Y(i-a,j-b)$$ (1)

In this case, the sign "X" stands for a filter’s dimensions, which are "a x b," "Y" stands for the input matrix, which is often an image, and "A" is the resultant feature map that is produced when the filter "X" is convolved with the input "Y." The symbol for this convolution operation is "X Y." The output of the convolutional layer is then subjected to a non-linear activation function (AF) after this convolutional procedure. The network becomes non-linear as a result of this AF. It is possible to process the feature map and introduce non-linearity while normalizing network data using a variety of non-linear activation functions. Sigmoid, hyperbolic tangent (Tanh), SoftMax, and rectified linear unit (ReLU) are some of these AFs. The ReLU activation function is used in this study, and it produces zero if the input is zero or less. Eq (2) refers to "ReLU" as the symbol for the mathematical representation of this ReLU process.


f(z)=max(0,z) (2)

**Fig 7:**
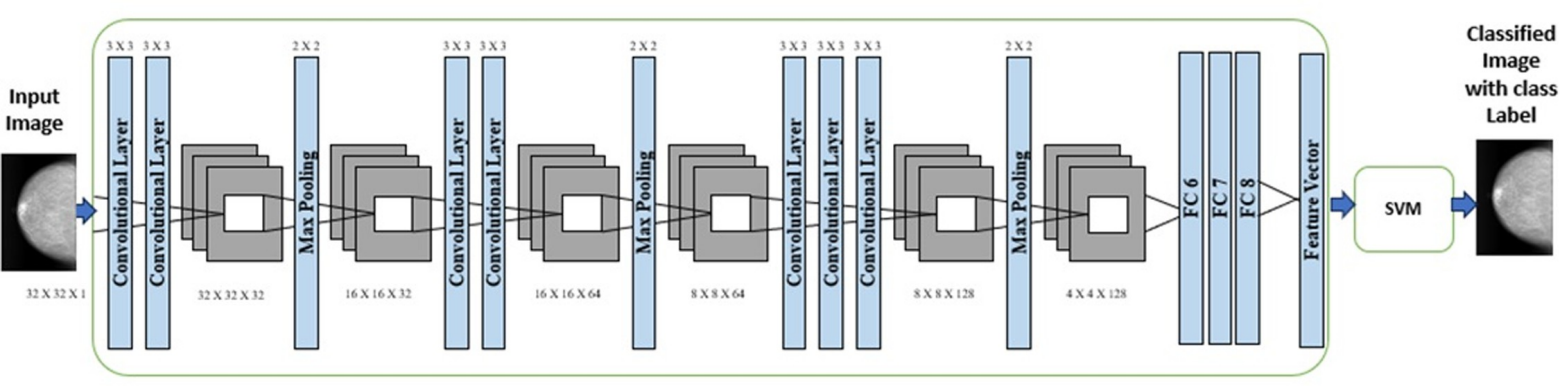
The simplified diagram of the customized AlexNet architecture.

**Fig 8:**
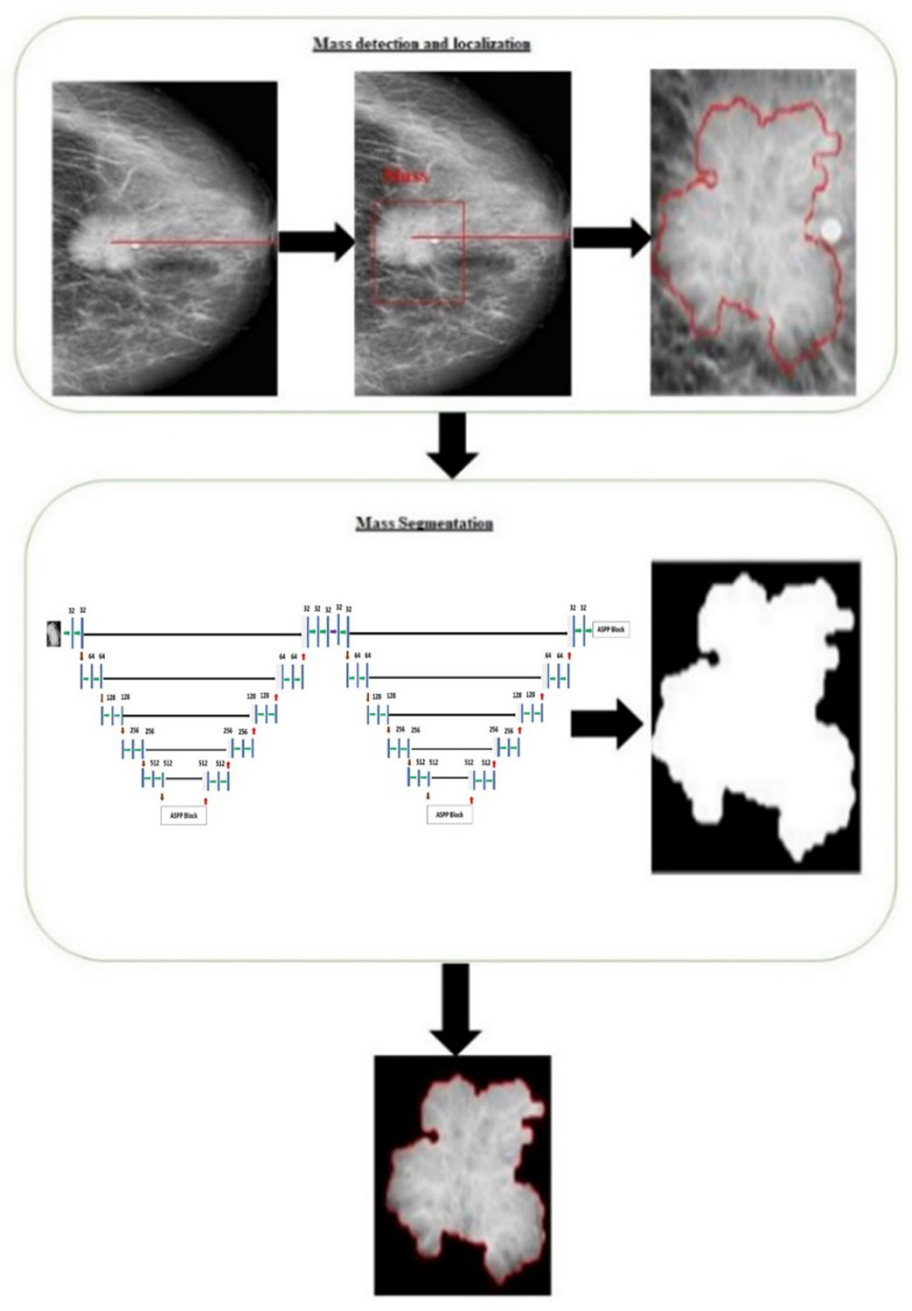
The suggested comprehensive framework.

In the realm of convolutional neural networks (CNNs), the pooling layer is commonly employed subsequent to the convolutional layer to decrease the dimensionality of the feature map while preserving essential features, often referred to as "down-sampling" in academic literature. Techniques such as average pooling, max-pooling, sum-pooling, and min-pooling are utilized by the pooling layer to reduce the dimensions of the activation map, retaining critical information. Before being forwarded to the fully connected layer, the feature map undergoes a flattening operation, converting the feature map matrix into a long vector as shown in Fig 8. In this specific application, 70% of pre-processed mammograms undergo convolutional operations in the convolutional layer. The proposed BreastNet-SVM comprises a total of thirteen layers, including three pooling layers, three fully connected layers, and seven convolutional layers, tailored for breast cancer identification, accommodating grayscale images of size 32 x 32 as shown in Fig 7. Initially, in the first two convolutional layers, 32 filters with a 3 x 3 kernel size are applied with the same padding, utilizing the ReLU activation function to introduce non-linearity. Following these layers, the original 32x32x32 image is down-sampled using a max-pooling layer with a 2x2 filter and stride of 2. Subsequently, two additional convolutional layers are employed, each featuring 64 filters, a 3x3 kernel, the same padding, and ReLU activation function. Post the initial max-pooling layer, which scales the image to 16x16x64, a second max-pooling layer with a 2x2 kernel size and stride further downsamples the image, resulting in an 8x8x64 image. The last three convolutional layers entail a total of 128 filters, each with a 3x3 kernel and ReLU activation. Following these layers, a third max-pooling layer is implemented, using a specific kernel size to reduce the input dimensions to a single vector sized 2048 x 1.

In the classification process using a convolutional neural network (CNN), the Fully Connected (FC) layer plays a crucial role after relevant features have been extracted. Serving as a bridge connecting neurons from the preceding layer to those above, the FC layer’s output is passed through an Activation Function (AF) to generate class scores for classification. Common techniques for classification tasks include Support Vector Machines (SVM) and SoftMax. In the BreastNet-SVM model, the support vector machine classifier is utilized to achieve optimal accuracy in distinguishing between benign and malignant breast cancer forms, with results evaluated at the performance layer. Deep learning tasks demand significant computational resources and training time, addressed through optimization algorithms like Stochastic Gradient Descent (SGD), adaptive moment estimation (Adam), and Root Mean Squared Propagation (RMSprop) to enhance performance. The Adam optimizer efficiently utilizes resources, RMSprop dynamically adjusts learning rates, and SGD utilizes model parameters and momentum to identify optimal parameters. Key metrics like accuracy and classification rate are evaluated at the performance layer to determine if the model meets learning criteria, requiring potential retraining. Upon training completion, the model and results are stored in the cloud for future use. During validation, the cloud-stored BreastNet-SVM model is retrieved for comparison with the trained model to assess performance. Utilizing a subset of the validation dataset, the previously trained model categorizes cases as "benign" or "malignant" based on cancer cell detection.

Our complete structure functions in a two-step sequential manner. It first recognizes and categorizes breast masses before going on to section these masses. Before beginning the intensive segmentation job, we take precautions by using a cutting-edge data augmentation technique. This method increases the dataset size of low-resolution mammograms while also enhancing their quality. To be clear, our novel design is specifically applied to the regions of interest (ROIs) containing breast masses that were determined in the preliminary stage. We used the YOLO model to identify breast abnormalities and distinguish between calcification and mass lesions in the earlier stage of our framework. The model thus produced bounding boxes around pertinent areas on the whole collection of mammograms. Nevertheless, the design is only used in the present phase on the ROIs associated with breast masses discovered earlier. It’s important to highlight that because calcification lesions lack exact reference annotations, this study only focuses on segmenting bulk lesions. The integrated YOLO model is used in the earliest stage of our framework to identify worrying breast lesions and distinguish between calcifications and mass lesions. Our newly developed architecture (shown in Fig 8) makes it easier to seamlessly transfer the areas of interest (ROIs) containing the identified masses to the following segmentation stage. Our method comprises expanding specific bounding box coordinates to cover more surrounding space around smaller tumors in order to account for the various sizes of breast masses. This generates a series of ROI images that are then downsized to 227 x 227 pixel dimensions since empirical research has shown that this size is the best input dimension for segmentation networks.

**Fig 9:**
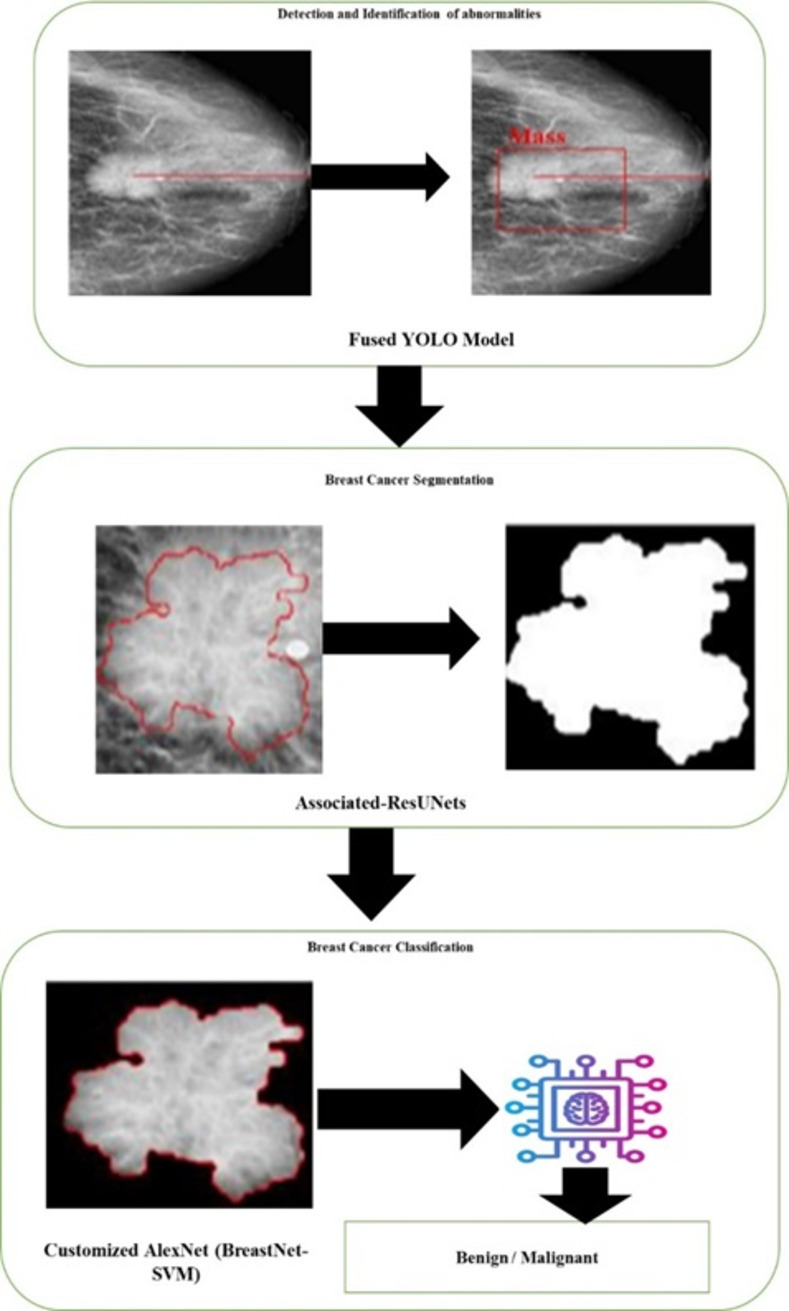
Proposed computer aided diagnosis framework.

The following steps helped us increase the system’s performance:

1. We began with a mammogram that had not been changed and had exact mass annotations highlighted in red. These annotations identified the mass’s Region of Interest (ROI).
2. We created a binary mask that successfully segmented the indicated mass to further fine-tune the procedure. This mask assisted in separating the bulk from the nearby tissue.
3. The segmented output of the mass, which now excluded the surrounding tissue, was obtained in the final stage. In the final classification phase, this segmented mass was used.
4. Using the segmented ROI masses as input, we trained a bespoke customized AlexNet (BreastNet-SVM) model for the classification task individually for each classification aim. We were able to forecast the pathology and categorize it as either benign or malignant as a result of this phase.
5. This completes our elaborate framework, which is seen in Fig 9. All automated procedures used in the evaluation and diagnosis of breast cancer are included.

## Experimental results

A. **Detection and identification**

For our YOLO model, we chose to concentrate on modifying a few key hyperparameters in order to streamline the process and highlight the most important factors. Mammography data was used in our trials, which involved randomly dividing it into 70% for training, 20% for testing, and 10% for validation for each class as shown in Table 1. We just changed the hyperparameters and kept the total trainable parameters constant throughout our studies.

**Table 1:** Data distribution of CBIS-DDSM dataset.

Dataset	Mass Data	ROIs Data	Augmented Data (ROIs x 6)	Training (70%)	Testing (20%)	Validation (10%)
CBIS- DDSM (Mass)	1468	1468	8808	6165	1761	882
CBIS- DDSM (Calcification)	1440	1440	8640	6048	1728	864
CBIS- DDSM (Architectural Distortion)	303	303	1818	1273	364	181
CBIS- DDSM (Normal)	162	162	972	680	194	98

To train our system for the recognition and categorization of breast lesions, we used CBIS-DDSM mammography dataset. We altered the model’s input data and adjusted Model2’s classification settings to enable multiple classes. Our findings unequivocally highlight the benefits of applying data augmentation and scaling methods to the original mammography dataset, with the dataset exhibiting especially notable performance gains. Notably, our model accomplished a greater rate of detection accuracy, which is an impressive feat. Since M2 was trained for both tasks using the enriched and scaled dataset, we ran tests where M1 was trained independently for Calcification and Mass detection. The results of these experimental trials are fully summarized in Table 2 below.

**Table 2:** Evaluation of the model’s performance across various prediction categories.

Dataset	M-1		M-2		
	Mass	Calcification	Mass	Calcification	Overall
CBIS-DDSM	97.9	89.9	96.2	88.7	93

In this study, a second assessment phase was added to evaluate the model for simultaneous detection and classification. This review procedure, which was extensive and included the integration of models developed under numerous situations, was explained in the preceding chapter. We first presented the results from the independent models, M-1 and M-2, utilizing the top-chosen mammograms from the enriched dataset to provide a thorough comprehension. Each pair of mammograms was evaluated together with six improved versions, such as rotated or transformed variations, of the original image. After carefully examining these sets, we chose the image with the best Intersection over Union (IoU) rating. The detection accuracy rate for each prediction class was then calculated after we integrated multiple models to form a new Fusion model, as shown in Table 3.

**Table 3:** Performance of fused model approach.

Dataset	Accuracy of detection (%)					
	M-1		M-2		Fused-model	
	mass	calcification	mass	calcification	mass	calcification
DDSM	97.9	89.9	96.2	88.7	98.5	93.4

The fused model achieved remarkable accuracy rates, notably 98.5%, when identifying mass lesions. This innovative fusion approach significantly enhanced the identification and classification of breast lesions. By achieving a detection accuracy rate of 98.5%, the fusion strategy effectively combined multiple models, delivering both speed and precision that surpassed current state-of-the-art methods. It’s worth highlighting that Architectural Distortion, in particular, exhibited outstanding diagnostic capabilities with a sensitivity of 95% for cancer patients and 93.09% for non-malignant cases as shown in Table 4.

**Table 4:** Performance in detection & classification on test dataset.

(Label)	(Precision)	(AUC)	(Sensitivity)	(Recall)	(Accuracy)
Normal	0.94	0.96	0.93	0.94	0.98
Architectural Distortion	0.95	0.95	0.95	0.95	0.98
Mass	0.94	0.95	0.93	0.94	0.96
Calcification	0.88	0.94	0.88	0.88	0.94

**Fig 10:**
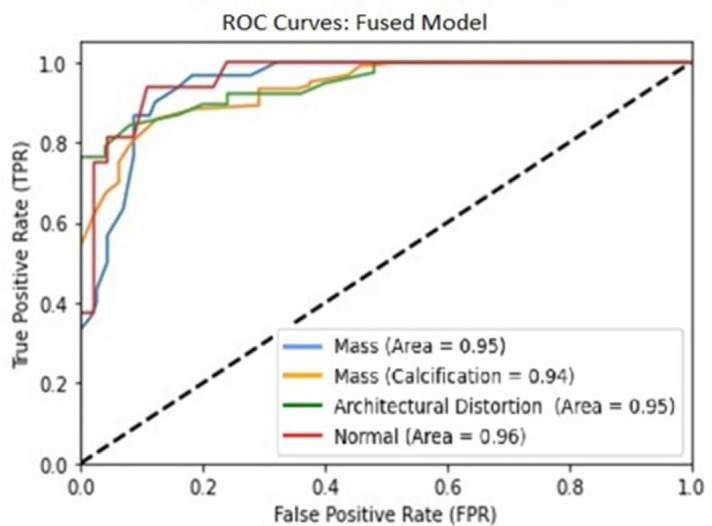
ROC curve proposed fused YOLO model on test sets.

Fig 10 shows the trade-off between FPR and TPR under various conditions using ROC curve plots. Particularly noteworthy were the excellent AUC scores of 0.95 for the Architectural Distortion and Mass cases and 0.96 for the Normal cases. The difficulty with calcification lesions comes from their variety of shapes and locations; they frequently appear as minor, irregular imperfections, making automated identification techniques work less efficiently.

B. **Mass segmentation**

Table 5 displays the evaluation findings for various testing sets, concentrating on the assessment of segmented maps at the per-pixel level. We determined two evaluation indicators for these results in this assessment.

**Table 5:** Segmentation performance on test set.

UNET Architectures	Dice-score (%)	IoU- score (%)
	CBIS-DDSM	
Standard UNet	89.88	86.44
Standard AUNet	90.26	88.03
Standard ResUNet	93.59	89.80
Associated-UNets	95.73	91.96
Associated-AUNets	95.83	92.18
Associated- ResUNets	95.89	92.28

The Associated-ResUNets architecture consistently outperforms the classic UNet, traditional AUNet, and ResUNet models, with considerable gains in Dice and IoU scores. Additionally, Associated-ResUNets and its variants exhibit great segmentation efficiency, with an average IOU score 92.28% and Dice score 95.89%.

C. **Classification using a BreastNet-SVM**

The study developed and assessed the BreastNet-SVM model using the publicly available CBIS-DDSM dataset. Multiple statistical criteria, including sensitivity, miss classification rate, specificity, and accuracy, were used to evaluate performance. These metrics established parameters for assessing the model’s overall performance and measured the model’s capacity to produce accurate predictions. They also helped to identify instances of wrong predictions. The following criteria have been established to evaluate the model’s performance.


$$Accuracy=\frac{\left(TN+TP\right)}{\left(TN+FP+FN+TP\right)}$$ (3)

$$Miss\;classificatio\;rate=\frac{\left(FN+FP\right)}{\left(TN+FP+FN+TP\right)}$$ (4)

$$Sensitivity=\frac{TP}{\left(TP+FN\right)}$$ (5)

$$Specificity=\frac{TN}{\left(TN+FP\right)}$$ (6)

BreastNet-SVM model for breast cancer diagnosis, this study tests three distinct optimizers (RMSprop, Adam, and SGD) on the CBIS-DDSM dataset. The efficiency of the model is then evaluated by a comparison analysis with more recent strategies. A comparison of the training phase, taking into account different input image sizes and optimizer selections, is presented in Table 6.

**Table 6:** Comparative evaluation of the BreastNet-SVM model while being trained.

Optimizer	Input image size	Specificity	Sensitivity	Accuracy	Misclassification rate
RMSprop	16×16	97.43%	90.04%	93.26%	6.74%
Adam	16×16	96.66%	93.34%	95.02%	4.98%
SGD	16×16	98.94%	97.93%	98.85%	1.15%
RMSprop	32×32	96.05%	99.74%	98.30%	1.70%
Adam	32×32	97.76%	95.53%	96.55%	3.45%
SGD	32×32	99.03%	98.60%	98.77%	1.23%
RMSprop	48×48	98.01%	96.99%	97.35%	2.65%
Adam	48×48	98.32%	98.34%	97.98%	2.02%
SGD	48×48	99.56%	98.78%	99.24%	0.76%

The study assessed the effectiveness of the BreastNet-SVM model for detecting breast cancer using three distinct optimizers (RMSprop, Adam, and SGD) and three different input image sizes (16x16, 32x32, and 48x48). The size of the input image and the optimizer selection were found to have a substantial impact on model performance. Notably, Adam and RMSprop both showed good performance, but the SGD optimizer consistently produced the maximum accuracy across all input sizes. Table 7 provides a comparative analysis during the study’s validation phase and summarizes these findings.

**Table 7:** Comparative evaluation of the suggested BreastNet-SVM model (validation).

Optimizer	Input image size	Specificity	Sensitivity	Accuracy	Misclassification rate
RMSprop	16×16	99.60%	79.49%	87.38%	12.62%
Adam	16×16	96.34%	90.24%	95.02%	4.98%
SGD	16×16	95.02%	97.09%	93.94%	6.06%
RMSprop	32×32	96.94%	87.98%	95.93%	4.07%
Adam	32×32	96.25%	94.03%	92.01%	7.99%
SGD	32×32	97.64%	96.04%	96.03%	3.97%
RMSprop	48×48	96.94%	94.11%	96.56%	3.44%
Adam	48×48	98.24%	95.45%	95.89%	4.11%
SGD	48×48	99.30%	97.13%	99.16%	0.84%

The efficiency of the BreastNet-SVM model for the detection of breast cancer varies depending on the optimizer chosen and the size of the input image. Especially with a 32x32 input image size, where it reached 99.16% accuracy, the SGD optimizer consistently produced the highest accuracy. Across a range of sizes, the Adam optimizer also worked admirably, whereas RMSprop showed great specificity but occasionally lower sensitivity. The setup of the model can be optimized for the identification of breast cancer using these performance indicators. A dataset of 6,165 samples that were divided into two categories malignant and benign was used to train the model. A confusion matrix was produced throughout the training process to evaluate its effectiveness as shown in Table 8.

**Table 8:** The BreastNet-SVM model’s best confusion matrix (training).

Actual Class	Predicted class	
	Benign	Malignant
Benign	2971	19
Malignant	47	3128

The BreastNet-SVM model in the study was trained using 2,990 samples from the benign category, and the performance of the model was assessed based on the accuracy of sample classification. The model accurately predicted 2,971 of these samples, however, 19 of them were misclassified. Using a dataset of 3,175 samples, the model was trained on malignant samples, accurately categorizing 3,128 samples while misclassifying 47. The validation phase of the BreastNet-SVM model’s confusion matrix, which corresponds to the SGD optimizer that produced the best accuracy, is shown in Table 9. In the validation phase, 882 samples in total were used to test the proposed model. These samples were then divided into two groups: malignant and benign.

**Table 9:** The BreastNet-SVM model’s best confusion matrix (validation).

Actual Class	Predicted class	
	Benign	Malignant
Benign	406	5
Malignant	10	461

**Fig 11:**
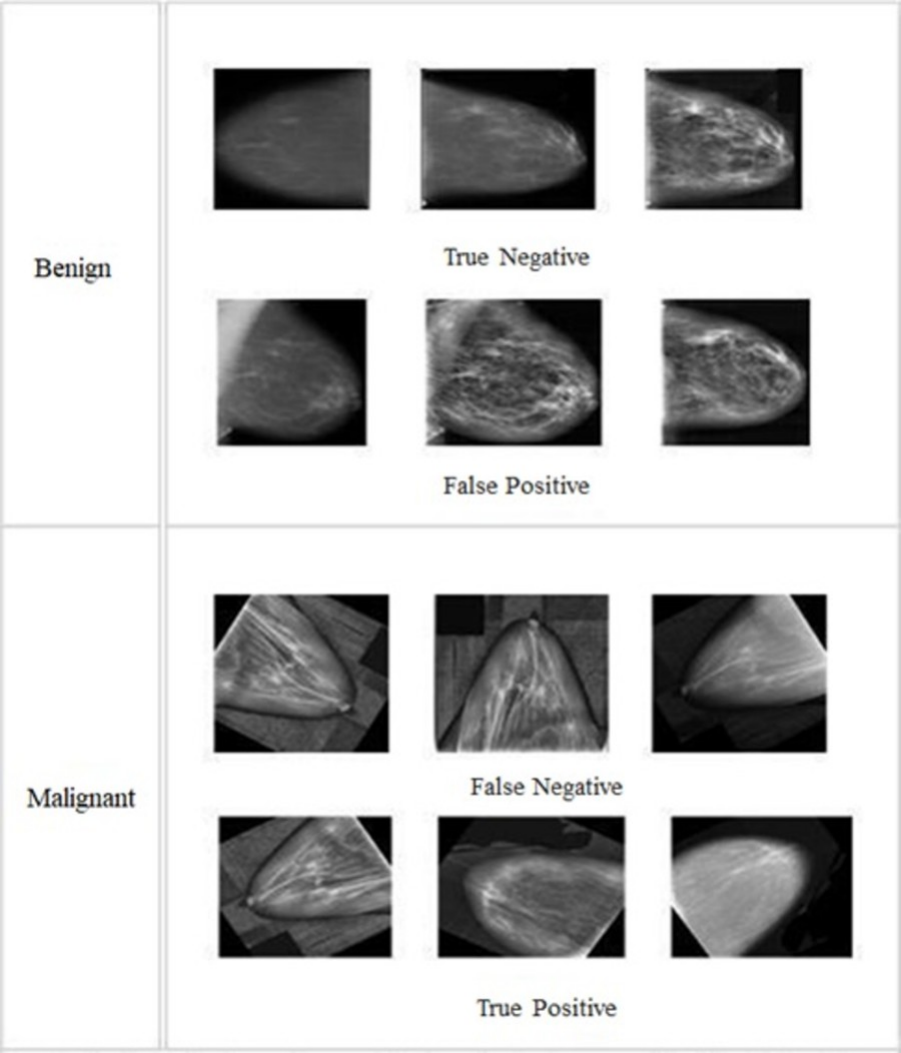
The BreastNet-SVM model’s benign and malignant breast tissues.

The suggested BreastNet-SVM model showed a high level of accuracy when predicting benign instances during the validation phase. The model accurately categorized 406 out of 411 benign samples, misclassifying only 5. Malignant samples required 471 samples for validation; the model correctly predicted 461 of them while mis prognosticating 10 of them. The findings of the improved AlexNet (BreastNet-SVM) model for detecting breast cancer are shown in Fig 11, including both benign and malignant outcomes. The right forecast of the first three images, which were classified as genuine negatives, shows how accurately the model classified benign instances. However, three images indicating cancer tissue were wrongly labeled as benign (false negatives), and another three images representing benign tissue were incorrectly labeled as malignant (false positives). The last three photos were appropriately classified as positive instances by the BreastNet-SVM model, which accurately reflected their malignancy status.

With a remarkable accuracy of 99.16%, the BreastNet-SVM model performed except**iona**lly well. Notably, it obtained an incredibly low misclassification rate of just 0.84%, the lowest percentage among comparable studies. Additionally, it demonstrated the highest sensitivity (97.13%) and specificity (99.30%) during the experimental analysis performed on the CBIS-DDSM dataset, which is available to the general public.

## Discussion

We conducted a comprehensive comparison of our proposed methodology with recent studies and similar methods. To ensure a thorough and equitable evaluation, we exclusively considered research that focused on the detection of Mass lesions, and these results are presented and contrasted in Table 10. When comparing the detection accuracy rates with other studies that utilized the CBIS-DDSM dataset, our fused YOLO models consistently outperformed in terms of overall performance.

**Table 10:** Evaluating the identification of mass lesions.

Author	Method	Dataset	Year	Detection accuracy rate (%)
Al-masni et al. [40]	YOLO	DDSM	2017	85.25
Agarwal et al. [41]	Mass probability map with CNN patch classifier	CBIS-DDSM	2019	82
Peng et al. [42]	Faster R-CNN	CBIS-DDSM	2020	93.45
Vedalankar et al. [43]	ALEX NET + SVM	DDSM	2021	92
Alruwailia et al. [44]	ResNet50	MIAS	2022	89.5
Das et al. [45]	Shallow convolutional neural network	DDSM	2023	80.4
Trang et al. [46]	CNN (X-ception, VGG16, ResNet-v2, ResNet50)	Private	2023	84.5
Ahmed S. Elkorany et al. [47]	CNN(Inception-V3, ResNet50, and AlexNet) + multiclass support vector machine (MSVM)	MIAS	2023	94.78
**Proposed Method**	**Fuesd YOLO Model**	**CBIS-DDSM**	**2023**	**98.5**

The BreastNet-SVM model delivered outstanding results, achieving an impressive accuracy of 99.16% as in Table 11. Notably, it demonstrated the lowest misclassification rate observed among similar studies in the field, standing at a mere 0.84%. Additionally, it exhibited the highest sensitivity at 97.13% and the highest specificity at 99.30% in the experimental analysis conducted using the publicly available CBIS-DDSM dataset.

**Table 11:** Comparison of the suggested method with cutting-edge techniques.

Author	Method	Dataset	Year	Accuracy (%)
Khan et al. [48]	GoogLeNet, VGGNet, and ResNet	LRH hospital Peshawar, Pakistan	2019	97.52
Moon et al. [49]	Ensemble (WA)	BUSI	2020	91.1
Saber et al. [50]	Inception-V2 ResNet, VGG-16, VGG-19, ResNet50, and Inception V3	MIAS	2021	98.96
Wang et al. [51]	Boosted EfficientNet model	Rectified Patch Camelyon (RPCam)	2021	96.5
Hekal et al. [52]	AlexNet and ResNet-50	CBIS-DDSM	2021	93.2
Alruwaili et al. [44]	ResNet50	MIAS	2022	89.5
Das et al. [45]	Shallow convolutional neural network	DDSM	2023	80.4
Rahman et al. [53]	ResNet50	INBreast	2023	93
**Proposed Method**	**Customized BreastNet-SVM**	**CBISDDSM**	**2023**	**99.16**

## Conclusion and future work

This research introduces an integrated deep learning-based CAD system aimed at assisting medical professionals in breast cancer diagnosis. The system comprises three key phases: detection, segmentation, and classification of breast abnormalities. The study demonstrates the effectiveness of various models and techniques, such as fused YOLO for simultaneous location and nature prediction, improved segmentation using attention mechanisms and residual blocks, and the integration of Associated-ResUNets and BreastNet-SVM) for accurate classification. The results highlight improved accuracy, reduced false positives/negatives, and the potential for broader medical imaging applications. Future research could expand this framework to incorporate more abnormalities and 3D medical images like CT scans and MRIs.

## References

[1] Siegel R. L., Miller K. D., Wagle N. S., and Jemal A., “Cancer statistics, 2023,” CA. Cancer J. Clin., vol. 73, no. 1, pp. 17–48, Jan. 2023, doi: 10.3322/caac.21763 36633525

[2] Duffy S. W. et al., “Mammography screening reduces rates of advanced and fatal breast cancers: Results in 549,091 women,” Cancer, vol. 126, no. 13, pp. 2971–2979, 2020, doi: 10.1002/cncr.32859 32390151 PMC7318598

[3] “Breast Cancer Overview: Causes, Symptoms, Signs, Stages & Types,” Cleveland Clinic. Accessed: Sep. 24, 2023. [Online]. Available: https://my.clevelandclinic.org/health/diseases/3986-breast-cancer.

[4] Doi K., “Diagnostic imaging over the last 50 years: research and development in medical imaging science and technology,” Phys. Med. Biol., vol. 51, no. 13, p. R5, Jun. 2006, doi: 10.1088/0031-9155/51/13/R02 16790920

[5] Fiorica J. V., “Breast Cancer Screening, Mammography, and Other Modalities,” Clin. Obstet. Gynecol., vol. 59, no. 4, p. 688, Dec. 2016, doi: 10.1097/GRF.0000000000000246 27741212

[6] Sasikala S., Bharathi M., Ezhilarasi M., Reddy M. R., and Arunkumar S., “Fusion of MLO and CC View Binary Patterns to Improve the Performance of Breast Cancer Diagnosis,” Curr. Med. Imaging, vol. 14, no. 4, pp. 651–658.

[7] Dembrower K. et al., “Effect of artificial intelligence-based triaging of breast cancer screening mammograms on cancer detection and radiologist workload: a retrospective simulation study,” Lancet Digit. Health, vol. 2, no. 9, pp. e468–e474, 2020. doi: 10.1016/S2589-7500(20)30185-0 33328114

[8] Couture H. D. et al., “Image analysis with deep learning to predict breast cancer grade, ER status, histologic subtype, and intrinsic subtype,” Npj Breast Cancer, vol. 4, no. 1, Art. no. 1, Sep. 2018, doi: 10.1038/s41523-018-0079-1 30182055 PMC6120869

[9] Celik Y., Talo M., Yildirim O., Karabatak M., and Acharya U. R., “Automated invasive ductal carcinoma detection based using deep transfer learning with whole-slide images,” Pattern Recognit. Lett., vol. 133, pp. 232–239, May 2020, doi: 10.1016/j.patrec.2020.03.011

[10] Rahman M. M., Ghasemi Y., Suley E., Zhou Y., Wang S., and Rogers J., “Machine Learning Based Computer Aided Diagnosis of Breast Cancer Utilizing Anthropometric and Clinical Features,” IRBM, vol. 42, no. 4, pp. 215–226, Aug. 2021, doi: 10.1016/j.irbm.2020.05.005

[11] Asgari Taghanaki S., Abhishek K., Cohen J. P., Cohen-Adad J., and Hamarneh G., “Deep semantic segmentation of natural and medical images: a review,” Artif. Intell. Rev., vol. 54, no. 1, pp. 137–178, Jan. 2021, doi: 10.1007/s10462-020-09854-1

[12] Mohebian M. R., Marateb H. R., Mansourian M., Mañanas M. A., and Mokarian F., “A Hybrid Computer-aided-diagnosis System for Prediction of Breast Cancer Recurrence (HPBCR) Using Optimized Ensemble Learning,” Comput. Struct. Biotechnol. J., vol. 15, pp. 75–85, 2017, doi: 10.1016/j.csbj.2016.11.004 28018557 PMC5173316

[13] Li H., Meng X., Wang T., Tang Y., and Yin Y., “Breast masses in mammography classification with local contour features,” Biomed. Eng. OnLine, vol. 16, no. 1, p. 44, Apr. 2017, doi: 10.1186/s12938-017-0332-0 28410616 PMC5391548

[14] Kriti, Virmani J., Dey N., and Kumar V., “PCA-PNN and PCA-SVM Based CAD Systems for Breast Density Classification,” in Applications of Intelligent Optimization in Biology and Medicine: Current Trends and Open Problems, Hassanien A.-E., Grosan C., and Fahmy Tolba M., Eds., in Intelligent Systems Reference Library. , Cham: Springer International Publishing, 2016, pp. 159–180. doi: 10.1007/978-3-319-21212-8_7

[15] Yassin N. I. R., Omran S., El Houby E. M. F., and Allam H., “Machine learning techniques for breast cancer computer aided diagnosis using different image modalities: A systematic review,” Comput. Methods Programs Biomed., vol. 156, pp. 25–45, Mar. 2018, doi: 10.1016/j.cmpb.2017.12.012 29428074

[16] Suzuki K., “Overview of deep learning in medical imaging,” Radiol. Phys. Technol., vol. 10, no. 3, pp. 257–273, Sep. 2017, doi: 10.1007/s12194-017-0406-5 28689314

[17] Qiu Y. et al., “A new approach to develop computer-aided diagnosis scheme of breast mass classification using deep learning technology,” J. X-Ray Sci. Technol., vol. 25, no. 5, pp. 751–763, Oct. 2017, doi: 10.3233/XST-16226 28436410 PMC5647205

[18] Yap M. H. et al., “Automated Breast Ultrasound Lesions Detection Using Convolutional Neural Networks,” IEEE J. Biomed. Health Inform., vol. 22, no. 4, pp. 1218–1226, Jul. 2018, doi: 10.1109/JBHI.2017.2731873 28796627

[19] Sun K. et al., “High-Resolution Representations for Labeling Pixels and Regions.” arXiv, Apr. 09, 2019. doi: 10.48550/arXiv.1904.04514

[20] Farabet C., Couprie C., Najman L., and LeCun Y., “Learning Hierarchical Features for Scene Labeling,” IEEE Trans. Pattern Anal. Mach. Intell., vol. 35, no. 8, pp. 1915–1929, Aug. 2013, doi: 10.1109/TPAMI.2012.231 23787344

[21] Eltrass A. S. and Salama M. S., “Fully automated scheme for computer-aided detection and breast cancer diagnosis using digitised mammograms,” IET Image Process., vol. 14, no. 3, pp. 495–505, 2020, doi: 10.1049/iet-ipr.2018.5953

[22] Siddiqui S. Y. et al., “Intelligent breast cancer prediction empowered with fusion and deep learning,” Comput. Mater. Contin., vol. 67, no. 1, pp. 1033–1049, 2021.

[23] Gardezi S. J. S., Elazab A., Lei B., and Wang T., “Breast Cancer Detection and Diagnosis Using Mammographic Data: Systematic Review,” J. Med. Internet Res., vol. 21, no. 7, p. e14464, Jul. 2019, doi: 10.2196/14464 31350843 PMC6688437

[24] Tavakoli N., Karimi M., Norouzi A., Karimi N., Samavi S., and Soroushmehr S. M. R., “Detection of abnormalities in mammograms using deep features,” J. Ambient Intell. Humaniz. Comput., vol. 14, no. 5, pp. 5355–5367, May 2023, doi: 10.1007/s12652-019-01639-x

[25] Moon W. K., Lee Y.-W., Ke H.-H., Lee S. H., Huang C.-S., and Chang R.-F., “Computer‐aided diagnosis of breast ultrasound images using ensemble learning from convolutional neural networks,” Comput. Methods Programs Biomed., vol. 190, p. 105361, Jul. 2020, doi: 10.1016/j.cmpb.2020.105361 32007839

[26] Khan S., Islam N., Jan Z., Ud Din I., and Rodrigues J. J. P. C., “A novel deep learning based framework for the detection and classification of breast cancer using transfer learning,” Pattern Recognit. Lett., vol. 125, pp. 1–6, Jul. 2019, doi: 10.1016/j.patrec.2019.03.022

[27] Peng J., Bao C., Hu C., Wang X., Jian W., and Liu W., “Automated mammographic mass detection using deformable convolution and multiscale features,” Med. Biol. Eng. Comput., vol. 58, no. 7, pp. 1405–1417, Jul. 2020, doi: 10.1007/s11517-020-02170-4 32297129

[28] Al-masni M. A. et al., “Detection and classification of the breast abnormalities in digital mammograms via regional Convolutional Neural Network,” in 2017 39th Annual International Conference of the IEEE Engineering in Medicine and Biology Society (EMBC), Seogwipo: IEEE, Jul. 2017, pp. 1230–1233. doi: 10.1109/EMBC.2017.8037053 29060098

[29] Haq A. U. et al., “3DCNN: Three-Layers Deep Convolutional Neural Network Architecture for Breast Cancer Detection using Clinical Image Data,” in 2020 17th International Computer Conference on Wavelet Active Media Technology and Information Processing (ICCWAMTIP), Chengdu, China: IEEE, Dec. 2020, pp. 83–88. doi: 10.1109/ICCWAMTIP51612.2020.9317312

[30] Vedalankar A. V., Gupta S. S., and Manthalkar R. R., “Addressing architectural distortion in mammogram using AlexNet and support vector machine,” Inform. Med. Unlocked, vol. 23, p. 100551, Jan. 2021, doi: 10.1016/j.imu.2021.100551

[31] Alruwaili M. and Gouda W., “Automated Breast Cancer Detection Models Based on Transfer Learning,” Sensors, vol. 22, no. 3, p. 876, Jan. 2022, doi: 10.3390/s22030876 35161622 PMC8838322

[32] Das H. S., Das A., Neog A., Mallik S., Bora K., and Zhao Z., “Breast cancer detection: Shallow convolutional neural network against deep convolutional neural networks based approach,” Front. Genet., vol. 13, 2023, Accessed: Sep. 22, 2023. [Online]. Available: doi: 10.3389/fgene.2022.1097207 36685963 PMC9846574

[33] Trang N. T. H., Long K. Q., An P. L., and Dang T. N., “Development of an Artificial Intelligence-Based Breast Cancer Detection Model by Combining Mammograms and Medical Health Records,” Diagnostics, vol. 13, no. 3, Art. no. 3, Jan. 2023, doi: 10.3390/diagnostics13030346 36766450 PMC9913958

[34] Hai J. et al., “Fully Convolutional DenseNet with Multiscale Context for Automated Breast Tumor Segmentation,” J. Healthc. Eng., vol. 2019, p. e8415485, Jan. 2019, doi: 10.1155/2019/8415485 30774849 PMC6350548

[35] Soulami K. B., Kaabouch N., Saidi M. N., and Tamtaoui A., “Breast cancer: One-stage automated detection, segmentation, and classification of digital mammograms using UNet model based-semantic segmentation,” Biomed. Signal Process. Control, vol. 66, p. 102481, Apr. 2021, doi: 10.1016/j.bspc.2021.102481

[36] Shams S., Platania R., Zhang J., Kim J., Lee K., and Park S.-J., “Deep Generative Breast Cancer Screening and Diagnosis,” in Medical Image Computing and Computer Assisted Intervention–MICCAI 2018, Frangi A. F., Schnabel J. A., Davatzikos C., Alberola-López C., and Fichtinger G., Eds., in Lecture Notes in Computer Science. Cham: Springer International Publishing, 2018, pp. 859–867. doi: 10.1007/978-3-030-00934-2_95

[37] Hekal A. A., Elnakib A., and Moustafa H. E.-D., “Automated early breast cancer detection and classification system,” Signal Image Video Process., vol. 15, no. 7, pp. 1497–1505, Oct. 2021, doi: 10.1007/s11760-021-01882-w

[38] Saber A., Sakr M., Abo-Seida O. M., Keshk A., and Chen H., “A Novel Deep-Learning Model for Automatic Detection and Classification of Breast Cancer Using the Transfer-Learning Technique,” IEEE Access, vol. 9, pp. 71194–71209, 2021, doi: 10.1109/ACCESS.2021.3079204

[39] Alam N., “Understanding YOLOv7 Neural Network,” Medium. Accessed: May 15, 2023. [Online]. Available: https://medium.com/@nahidalam/understanding-yolov7-neural-network-343889e32e4e.

[40] Al-masni M. A. et al., “Detection and classification of the breast abnormalities in digital mammograms via regional Convolutional Neural Network,” in 2017 39th Annual International Conference of the IEEE Engineering in Medicine and Biology Society (EMBC), Jul. 2017, pp. 1230–1233. doi: 10.1109/EMBC.2017.8037053 29060098

[41] Agarwal R., Diaz O., Lladó X., Yap M. H., and Martí R., “Automatic mass detection in mammograms using deep convolutional neural networks,” J. Med. Imaging, vol. 6, no. 03, p. 1, Feb. 2019, doi: 10.1117/1.JMI.6.3.031409 35834317 PMC6381602

[42] Peng J., Bao C., Hu C., Wang X., Jian W., and Liu W., “Automated mammographic mass detection using deformable convolution and multiscale features,” Med. Biol. Eng. Comput., vol. 58, no. 7, pp. 1405–1417, Jul. 2020, doi: 10.1007/s11517-020-02170-4 32297129

[43] Vedalankar A. V., Gupta S. S., and Manthalkar R. R., “Addressing architectural distortion in mammogram using AlexNet and support vector machine,” Inform. Med. Unlocked, vol. 23, p. 100551, 2021, doi: 10.1016/j.imu.2021.100551

[44] Alruwaili M. and Gouda W., “Automated Breast Cancer Detection Models Based on Transfer Learning,” Sensors, vol. 22, no. 3, p. 876, Jan. 2022, doi: 10.3390/s22030876 35161622 PMC8838322

[45] Das H. S., Das A., Neog A., Mallik S., Bora K., and Zhao Z., “Breast cancer detection: Shallow convolutional neural network against deep convolutional neural networks based approach,” Front. Genet., vol. 13, p. 1097207, Jan. 2023, doi: 10.3389/fgene.2022.1097207 36685963 PMC9846574

[46] Trang N. T. H., Long K. Q., An P. L., and Dang T. N., “Development of an Artificial Intelligence-Based Breast Cancer Detection Model by Combining Mammograms and Medical Health Records,” Diagnostics, vol. 13, no. 3, p. 346, Jan. 2023, doi: 10.3390/diagnostics13030346 36766450 PMC9913958

[47] Elkorany A. S. and Elsharkawy Z. F., “Efficient breast cancer mammograms diagnosis using three deep neural networks and term variance,” Sci. Rep., vol. 13, no. 1, p. 2663, Feb. 2023, doi: 10.1038/s41598-023-29875-4 36792720 PMC9932150

[48] Khan S., Islam N., Jan Z., Ud Din I., and Rodrigues J. J. P. C., “A novel deep learning based framework for the detection and classification of breast cancer using transfer learning,” Pattern Recognit. Lett., vol. 125, pp. 1–6, Jul. 2019, doi: 10.1016/j.patrec.2019.03.022

[49] Moon W. K., Lee Y.-W., Ke H.-H., Lee S. H., Huang C.-S., and Chang R.-F., “Computer‐aided diagnosis of breast ultrasound images using ensemble learning from convolutional neural networks,” Comput. Methods Programs Biomed., vol. 190, p. 105361, Jul. 2020, doi: 10.1016/j.cmpb.2020.105361 32007839

[50] Saber A., Sakr M., Abo-Seida O. M., Keshk A., and Chen H., “A Novel Deep-Learning Model for Automatic Detection and Classification of Breast Cancer Using the Transfer-Learning Technique,” IEEE Access, vol. 9, pp. 71194–71209, 2021, doi: 10.1109/ACCESS.2021.3079204

[51] Wang J., Liu Q., Xie H., Yang Z., and Zhou H., “Boosted EfficientNet: Detection of Lymph Node Metastases in Breast Cancer Using Convolutional Neural Networks,” Cancers, vol. 13, no. 4, p. 661, Feb. 2021, doi: 10.3390/cancers13040661 33562232 PMC7915222

[52] Hekal A. A., Elnakib A., and Moustafa H. E.-D., “Automated early breast cancer detection and classification system,” Signal Image Video Process., vol. 15, no. 7, pp. 1497–1505, Oct. 2021, doi: 10.1007/s11760-021-01882-w

[53] Rahman H., Naik Bukht T. F., Ahmad R., Almadhor A., and Javed A. R., “Efficient Breast Cancer Diagnosis from Complex Mammographic Images Using Deep Convolutional Neural Network,” Comput. Intell. Neurosci., vol. 2023, pp. 1–11, Mar. 2023, doi: 10.1155/2023/7717712 36909966 PMC9998154

